# A Theoretical Analysis of the Preventive Role of English as a Foreign Language Teachers’ Occupational Adjustment and Psychological Hardiness in Their Job Burnout

**DOI:** 10.3389/fpsyg.2022.939617

**Published:** 2022-06-14

**Authors:** Xiaoying Li

**Affiliations:** School of Public Policy and Management, Guangxi University, Nanning, China

**Keywords:** occupational adjustment, psychological hardiness, burnout, EFL teacher, L2 education

## Abstract

Teaching has long been considered as one of the most challenging professions worldwide due to the involvement of numerous linguistic, socio-political, social, and psych-emotional factors. Because of these factors, english as a foreign language (EFL) teachers are psychologically, emotionally, and physically pressed in their job. These damaging pressures often result in negative academic outcomes for teachers, students, and educational systems. Despite dire consequences, few studies (if any) have examined the role of psycho-emotional factors in improving teaching performance and reducing negative emotions at the workplace. To fill this gap, this mini-review article aimed to present a theoretical analysis of three constructs of occupational adjustment, psychological hardiness, and burnout. In so doing, the definitions, conceptualizations, dimensions, and empirical studies related to each variable were presented. The study ends with the existing research gaps and offers some implications for EFL teachers and other stakeholders by increasing their knowledge of emotions in occupational environments and the consequences that such emotions can have for an organization.

## Introduction

It is widely believed that human resources are the most important capitals and elements of a successful organization and determine its quality, survival, and improvement (Bianchi et al., 2015; Vaezi et al., 2016). Teaching as one of the most demanding and stressful professions in the world is not an exception and qualified teachers are vital for academic success (Coombe, 2008; Mercer, 2020; Greenier et al., 2021). As teaching is critical for architecting societies, many unwanted tensions, stresses, apprehensions, and other psych-emotional pressures are placed on teachers leading to numerous consequences for teachers, students, and educational systems (Johnson et al., 2005; Wang, 2021). To keep up with these adversities, english as a foreign language (EFL) teachers require high levels of intellectual, physical, and emotional strengths (Davaribina and Asl, 2021). Moreover, as second/foreign language education is a relational job involving several social, psychological, and emotional interactions (Strachan, 2020), EFL teachers must have occupational adjustment (OA, hereafter) as well. The concept of OA refers to those behaviors that produce a good performance and a positive attitude toward one’s role in a profession (Rahimi-Dadkan and Nastiezaie, 2016). It is a complex construct comprising an employee’s perceived sense of achievement, altruism, safety, autonomy, comfort, status, and adjustment (Coetzee and Stoltz, 2015). Research indicates that OA influences teachers’ job satisfaction and performance and reduces their stress, tension, and occupational conflicts (Rahimi-Dadkan and Nastiezaie, 2016).

Another area that OA can facilitate the ground for is EFL teachers’ psychological hardiness as a growing line of research (Quick et al., 2013). To survive in one of the five most difficult jobs, EFL teachers must be psychologically hard in the face of challenges overwhelming L2 education (Judkins et al., 2020). Simply, psychological hardiness pertains to one’s ability to manage and respond to stressful events by means of suitable coping strategies that transfigure tough situations into learning chances (Maddi, 2004). Therefore, the term can be seen as a mindset through which an individual perceives obstacles, problems, and stressful conditions as personal challenges rather than road barriers of development. Research corroborates that psychological hardiness affects EFL teachers’ pedagogical performance under pressure, motivation, affectivity, optimism, decisiveness, perfectionism, and resilience (Cole et al., 2004; Erkutlu, 2011; Tokhmehforoushan Khiabani and Hadidi Tamjid, 2017; Tarajová and Metruk, 2020).

Both OA and psychological hardiness can prevent job burnout as teachers’ high adaptability to a working environment and capacity to cope with setbacks minimize the danger of emotional exhaustion and job-leaving rate. Burnout is a common feature of novice teachers who are at the beginning of their teaching career (Heidari and Gorjian, 2017). According to Maslach (1976), burnout is “a syndrome of emotional exhaustion, lack of personal accomplishments, and depersonalization and the fire of enthusiasm and commitment to success being reduced to ashes” (p. 1). It also refers to negative changes in one’s attitude, mood, and behavior when facing occupational stress (Mede, 2009). Burnout can be the consequence of constant changes in educational policies, imbalance between course demands and goals, limited instructional time, low salary, and tense working climate (Sadeghi and Khezrlou, 2014; Heidari and Gorjian, 2017). This negative construct can exert many detrimental impacts on teachers’ performance and effectiveness (Schaufeli et al., 2008). It also diminishes their job satisfaction, tolerance, sympathy, and quality of service (Maslach and Jackson, 1984; Piko, 2006). Despite these promising findings, the preventive role of OA and psychological hardiness in reducing or preventing EFL teachers’ burnout level has been overlooked, to date. To fill this gap, this mini-review article aimed to present a theoretical analysis of these three influential constructs referring to their underpinnings, definitions, and related studies.

## Background

### The Definition of Occupational Adjustment

As a concept related to organizational settings, OA is defined as the degree of compatibility between a person and a workplace (Kuerer et al., 2007). It contains one’s behaviors at work that bring about good performance and create positive attitudes toward a job (Rahimi-Dadkan and Nastiezaie, 2016). In layman terms, OA or work adjustment concerns one’s abilities, interests, and tendencies to be compatible with a specific job. It shows itself *via* the degree of satisfaction that a person represents in a workplace.

### The Theory of Work Adjustment

Work Adjustment Theory (WAT) is a theory of satisfaction and needs that explicates how and why employers adjust to their workplace surroundings (Dawis and Lofquist, 1984). WAT is one of the most popular and validated theories in vocational psychology (Eggerth, 2008) which is the outcome of a person’s (P) interaction with environment (E). The theory argues that an employee/individual would act on environmental stimuli or vice versa (Dawis, 2005). It considers work as an interactive and mutual process between P and E (Dawis and Lofquist, 1984). Furthermore, based on this theory, an individual’s degree of job satisfaction and adjustment hinges on P variables, E variables, their combination (Dawis, 2005). Therefore, in WAT, adjustment to an occupation can be explained by a satisfaction that drives professional behavior and the correspondence between P and E factors. In addition to enhancing job satisfaction, efficiency, quality, and performance, work adjustment or OA can spread the seeds of psychological hardiness in people in that when they have high levels of adaptability and compatibility with their workplace and colleagues they manifest more toughness in the face of challenges and hardly get burned out.

### The Conceptualization of Psychological Hardiness

The construct of psychological hardiness can be explained as a personal capability to positively and confidently react to negative happenings and challenges in one’s life and profession (Maddi, 2004). For Hiver and Dörnyei (2017), hardiness is a person’s competence to combat against and diminish the negative impacts of stress on his/her performance. It encompasses three components of *commitment*, *control*, and *challenge* (Sheard and Golby, 2010). By commitment, Sheard and Golby (2010) meant the tendency to intensely engage oneself in a work or task. Likewise, control refers to a person’s proclivity to recognize and perform as though he/she has an active role in diverse life occurrences and is by no means helpless and passive. Finally, they described challenge as the idea that change is prevalent in one’s life and its anticipation is beneficial for personal development. These elements perfectly reflect the essence of L2 education in which EFL teachers and students have to handle numerous linguistic, intercultural, and psycho-emotional factors simultaneously. To perform best in such a challenging job, EFL teachers need ample pedagogical knowledge and psychological hardiness to convert setbacks to learning opportunities for students.

### The Cognates of Psychological Hardiness

In the pertinent literature, there have been proposed and used some similar concepts related to psychological hardiness which convey similar but not absolutely identical messages. They encompass *coping*, *immunity*, *resilience*, and *buoyancy*. By definition, coping has do to with particular techniques and strategies that a person utilizes to resolve problems (Somerfield and McCrae, 2000). Moreover, immunity is a defensive mechanism that an individual constitutes and uses to prevent setbacks, adversities, and damages to his/her motivation, identity, and practice (Hiver, 2017). Furthermore, resilience concerns one’s capability to identify and fight back general life challenges and adversities, though buoyancy is more specific and has to do with academic adversities and difficulties (Martin and Marsh, 2019). Despite their different grounds, these cognate terms do not have clear-cut boundaries and they are still, in many cases, interchangeably used in educational research and practice.

### Job Burnout: Definitions, Sources, and Symptoms

The concept of burnout was first introduced by Freudenberger (1974) in the context of social workers. It is one of the main occupational problems typically realized as a reaction to job and organizational pressures (Hajihasani, 2017). The term has been defined by different scholars who unanimously regarded it as a psychological syndrome. For instance, Maslach and Jackson (1982) described burnout as a syndrome of emotional exhaustion, depersonalization, and reduced personal accomplishment. Likewise, Swider and Zimmerman (2010) considered it as a psychological syndrome that affects one’s work, responsibilities, clients, family, friends, and themselves. Useche et al. (2015) saw burnout as a reaction to the chronic exposure to occupational stressors. Furthermore, Anand and Monika (2017) defined the term as a maladaptive psycho physiological and behavioral reaction to job-related stressors.

Concerning the sources and origins of teacher burnout, several studies have been done whose results indicate that this serious problem is the consequence of teachers’ perceptions of interpersonal factors, school climate, stakeholders’ relations, administrative climate, lack of autonomy, demotivating factors (low salary, low student motivation, low social status), political interference, discrimination, teaching anxiety, and lack of administrative support (Nadeem et al., 2011; Soodmand Afshar and Doosti, 2015; Gürbüz and Dede, 2018; Perrone et al., 2019; Pressley, 2021, among others). The most common symptoms of this negative construct are inadequate energy, reduced patience, increased suspicion and pessimism, lack of passion and enthusiasm toward a job, occupational stress, emotional unresponsiveness, and lack of compliance with the job (Ahola and Hakanen, 2007; Takahashi, 2016).

### Coping Strategies for Teacher Burnout

To mitigate this aversive emotion in teachers, several strategies have been proposed through which EFL teachers can stop and minimize the impact of burnout on their career. They include: establishing a positive classroom rapport with students, class management, staff meetings, reflection, sustaining resilience and optimism, optimizing school climate, supportive administrative attitudes, agency in decision-making, community support, intrinsic motivation, and using technologies in the class (Chen, 2010; Larrivee, 2012; Gruenert and Whitaker, 2015; Akbari and Eghtesadi, 2017; Atmaca, 2017).

### Related Studies

Reviewing the literature, researchers can identify that OA has been empirically studied in relation to variables such as psychological empowerment, burnout, work-related conflicts, job satisfaction, performance, and work stress (Wen-e, 2000; Rahimi-Dadkan and Nastiezaie, 2016). However, the impact of OA on EFL teachers’ career and performance has been ignored, to date. One of the areas that L2 researches can run complementary studies is the association between OA and psychological hardiness of EFL teachers. The construct of psychological has witness more empirical research in comparison to OA signifying that it can enhance teachers’ instructional performance, motivation, affectivity, perfectionism, resilience, optimism, decisiveness, and reduce their burnout level (Cole et al., 2004; Erkutlu, 2011; Tokhmehforoushan Khiabani and Hadidi Tamjid, 2017; Tarajová and Metruk, 2020). As for teachers’ burnout, research demonstrates that it negatively influences their satisfaction level, performance, commitment, enthusiasm, self-esteem, self-efficacy, teaching quality, and so forth (Kyriacou, 2001; Ghanizadeh and Ghonsooly, 2014; Colomeischi, 2015; Akhavanattar and Ahmadi, 2017; Heidari and Gorjian, 2017). Moreover, it has been identified that social participation, emotional intelligence, strong mental wellbeing, and positive emotions can prevent teacher burnout (Mahmoodi and Ghaslani, 2014; Herman et al., 2018; Capone et al., 2019). What is under-researched in this line of inquiry is the preventive role of OA and psychological hardiness of EFL teachers in blocking or decreasing their sense of burnout. Most of the existing studies are simple correlational studies and the developmental paths of these three constructs have been ignored. Hence, running qualitative case studies can provide more clear images of how these variables are shaped and work in the context of L2 education.

## Concluding Remarks

In this mini-review, it was argued that having OA and psychological hardiness can stop or reduce EFL teachers’ burnout level as a personal and social problem that can damage many educational efforts. It was also pinpointed that by facilitating the ground for teachers to develop their adaptability and adjustment skills in workplaces, EFL teachers’ mental strengths to handle L2 complications and teaching performance boost exponentially. The interplay of the variables in this review is justifiable because when a teacher adjusts easily and efficiently to an occupational context, he/she manages and understands the challenges of that job better and becomes psychologically hard in facing adversities. Hence, the likelihood of becoming burned out meaningfully falls down. Drawing on these propositions, this article can have implications for EFL teachers, teacher trainers, human resources management, and researchers. EFL teachers can use this study as a starting point to realize the power of psycho-emotional factors in remaining, acclimatizing, and improving in their teaching career. Their understanding and use of strategies to enhance their OA and psychological hardiness can increase as well. Moreover, teacher trainers can use this article to design professional development courses for EFL teachers to cultivate practical ways of having and sustaining OA and psychological hardiness in L2 education. Likewise, human resources management may benefit from this study in that they can think of and develop strategies and methods to reduce teacher burnout levels, especially through empowering them with psycho-emotional trainings. Finally, L2 researchers may find this article of value in that it sparked some light on the role of emotions in workplaces and their quality. It reviewed the existing studies showing the gaps in this domain. For instance, qualitative and mixed-methods research designs have been rarely employed to unpack the relationship between psycho-emotional variables in L2 teaching profession. Likewise, future researchers can conduct phenomenological research on how OA and psychological hardiness prevents negative constructs like burnout, pessimism, boredom, demotivation, and the like. The association between interpersonal communication skills and the three variables reviewed in this article is also a fresh idea for research. Lastly, the role of cultural factors and contextual variation can also be examined in relation to work-related constructs.

## Author Contributions

The author confirms being the sole contributor of this work and has approved it for publication.

## Conflict of Interest

The author declares that the research was conducted in the absence of any commercial or financial relationships that could be construed as a potential conflict of interest.

## Publisher’s Note

All claims expressed in this article are solely those of the authors and do not necessarily represent those of their affiliated organizations, or those of the publisher, the editors and the reviewers. Any product that may be evaluated in this article, or claim that may be made by its manufacturer, is not guaranteed or endorsed by the publisher.
